# Genetic effects of rs3740199 polymorphism in *ADAM12* gene on knee osteoarthritis: a meta-analysis

**DOI:** 10.1186/s13018-017-0594-z

**Published:** 2017-06-20

**Authors:** Zheng Hao, Xin Li, Jin Dai, Baocheng Zhao, Qing Jiang

**Affiliations:** 1 Center of Diagnosis and Treatment for Developmental Dysplasia of the Hip, Nanjing Zhongyangmen Community Health Service Center, Kang’ai Hospital, Nanjing, 210037 Jiangsu People’s Republic of China; 2 Department of HIV/AIDS/STI Prevention and Control, Nanjing Municipal Center for Diseases Control and Prevention, Nanjing, 210009 Jiangsu People’s Republic of China; 3 Department of Sports Medicine and Adult Reconstructive Surgery, Drum Tower Hospital, School of Medicine, Nanjing University, 321 Zhongshan Road, Nanjing, 210008 Jiangsu People’s Republic of China; 4 Laboratory for Bone and Joint Disease, Model Animal Research Center (MARC), Nanjing University, Nanjing, 210093 Jiangsu China

**Keywords:** Osteoarthritis, *ADAM12*, rs3740199, Polymorphism, Meta-analysis

## Abstract

**Background:**

Knee osteoarthritis (OA) is a complex arthritic condition in which genetic factors play an important role. *ADAM12* gene is one of the recognized candidate genes although the results are conflicting. To derive a more precise estimation of the association between rs3740199 polymorphism in *ADAM12* gene and risk of knee OA, we performed a meta-analysis based on six related studies, including a total of 2185 cases and 3716 controls.

**Methods:**

A comprehensive search was performed to identify related studies up to April 14, 2017. We used odds ratios (ORs) with 95% confidence intervals (CIs) to assess the strength of the association. Different genetic models were used to assess the pooled and stratified data.

**Results:**

Overall, no significant association was found in all genetic models (C vs. G, OR = 0.983, 95% CI = 0.910–1.061; CC vs. GG, OR = 1.033, 95% CI = 0.851–1.255; CG vs. GG, OR = 1.030, 95% CI = 0.877–1.209; CC/CG vs. GG, OR = 1.031, 95% CI = 0.886–1.201; CC vs. CG/GG, OR = 1.017, 95% CI = 0.868–1.190). When stratified by ethnicity, no significant association was found.

**Conclusions:**

This meta-analysis suggested that the rs3740199 polymorphism does not contribute to the development of knee OA. Additional well-designed large studies are required to confirm these findings in different populations.

## Background

Osteoarthritis (OA) is a complex arthritic condition characterized by progressive cartilage loss, synovitis, osteophyte formation, and subchondral sclerosis. It is a cause of important handicap among the elderly [1, 2]. It has been reported that there were nearly 85 million OA patients in the world in 2009, and it might increase to 122 million in 2017. Hence, it is an enormous burden on the national economy and healthcare system [3, 4]. OA is a multifactorial disease resulting from the combined influence of environmental factors and genes [5]. Age, joint injury, and obesity are the major risk factors [6, 7]. Several studies have identified some genetic factors such as *ASPN* [8], *FRZB* [9], and *GDF5* [10]. These three genes are involved in controlling growth and differentiation pathways [11]. Many other polymorphisms have shown association to OA although the results are inconsistent. Further research is needed to replicate these findings and identify some new genetic factors [12].

A disintegrin and metalloprotease (ADAM), a member of the Zn-dependent metzincin superfamily, is associated with many complex diseases such as heart disease, rheumatoid arthritis, Alzheimer’s disease, and cancer [13, 14]. *ADAM12* may play an important role in chondrocyte proliferation, maturation, bone formation, and osteoclast differentiation [15–18]. ADAM12 is up-regulated in multinucleated giant cells surrounding loose hip implants and OA cartilage [19, 20]. Recently, promising but contradictory data have been published for the association of *ADAM12* with OA [21–25]. Poonpet et al*.* [24] and Kerna et al. [25] found that rs3740199 in *ADAM12* was associated with knee OA risk although the results were conflicting rather than conclusion [12, 23, 26, 27].

In the present study, a meta-analysis was performed to determine the overall association between *ADAM12* rs3740199 polymorphism and knee OA susceptibility and whether the association varies by ethnicity.

## Methods

### Literature search strategy

To identify all relevant reports on rs3740199 polymorphism and knee OA risk, we performed a systematic search for all English language papers from PubMed (the last search update was April 14, 2017), using the key words “rs3740199” or “*ADAM12*,” “polymorphism” or “polymorphisms” or “SNP,” “osteoarthritis” or “OA”. Additional eligible studies were identified by a manual search of the references of retrieved studies and review articles.

According to the following criteria, six studies were included in this meta-analysis: (1) was a cohort or case-control study; (2) was a study of the *ADAM12* rs3740199 polymorphism and knee OA risk; and (3) contained available genotype or allele frequency of rs3740199.

### Data extraction

Two of the investigators extracted all data independently according to the criteria described above. We developed a data extraction sheet including year of publication, the first author’s name, OA type, country of origin, ethnicity, assessment of OA, genotyping method, source of control groups, genotype, and allele frequency. For studies contain the results from different knee OA types, each type was treated independently. Any controversies of the data were discussed within our research team and the authors reached a consensus on all items.

### Statistical methods

Allele frequencies of the *ADAM12* rs3740199 polymorphism from the six eligible studies were calculated by the allele counting method respectively. Hardy–Weinberg equilibrium (HWE) was used to evaluate the deviation of data associated with the *ADAM12* rs3740199 SNP in the control groups using *χ* ^2^ test. The strength of association between the *ADAM12* rs3740199 polymorphism and knee OA susceptibility was evaluated by pooled odds ratios (ORs) and their 95% confidence intervals (CIs). The significance of the ORs and 95% CIs was determined by *Z* test. The pooled ORs and 95% CIs were performed for additive model (C vs. G), co-dominant model (CC vs. GG; CG vs. GG), dominant model (CC/CG vs. GG), and recessive model (CC vs. CG/GG). Stratified analysis was also performed by ethnicity.

We assessed the between-study heterogeneity using chi-square-based *Q* test. If the *P* value was less than 0.10, the heterogeneity was considered significant. We also used the *I* ^2^ statistic (*I* ^2^ = 100% × (*Q* − *df*)/Q) to quantify heterogeneity. *I* ^2^ greater than 50% indicated the presence of heterogeneity among studies. The fixed-effects model based on the Mantel–Haenszel method and the random-effects model based on the Dersimonian and Laird method were used to pool the data [28]. The random-effects model was more appropriate in the presence of heterogeneity; otherwise, the two methods provide similar results.

In meta-analysis, publication bias is also a concern. To test for publication bias, both Egger’s and Begg’s test are commonly used [29]. In this study, publication bias was evaluated by funnel plot and the linear regression asymmetry test.

All analyses were carried out using Stata software version 8.2 (Stata Corporation, College Station, TX, USA). All tests were two-sided.

## Results

### Characteristics of the included studies

**Fig. 1:**
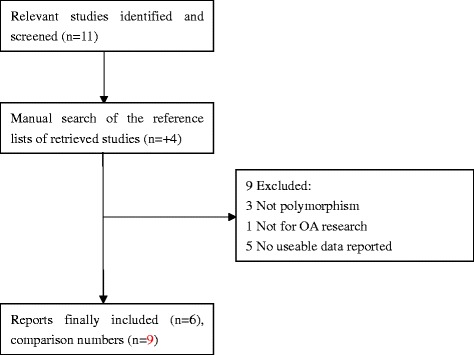
Studies identified with criteria for inclusion and exclusion

Eleven relevant studies identified and screened. Four studies were added through manual search of the reference lists of retrieved studies. Nine of the 15 studies were excluded: three not polymorphism, one not for OA research, and five no useable data reported. A total of six reports were identified [12, 23–27]. Among these, Rodriguez-Lopez et al. reported six sample collections [12] while Kerna et al. included subjects with tibiofemoral knee OA (TFOA) and patellofemoral knee OA (PFOA) [25], they were considered as independent studies. Finally, nine studies with 2185 cases and 3716 controls were included in the present meta-analysis. The detailed study flow chart was illustrated in Fig. 1. Characteristics of the nine studies were listed in Tables 1 and 2. Of eligible studies, four and five studies were conducted in Asian and European populations respectively.

**Table 1:** Characteristics of literatures included in this meta-analysis

Year	First author	OA type	Country	Ethnicity	Assessment of OA	Genetyping	Source of controls	Cases	Controls
2009	J. Rodriguez-Lopez	TKR	Spain	European	K/L score	Multiplex single-base extension	PB	262	294
		TKR	UK	European	K/L score	Multiplex single-base extension	PB	360	698
		TKR	Greece	European	K/L score	Multiplex single-base extension	PB	159	193
2009	I. Kerna	TFOA	Estonian	European	OA score	PCR-RFLP	PB	66	123
		PFOA	Estonian	European	OA score	PCR-RFLP	PB	97	92
2012	Min-Ho Shin	Knee OA	Korea	Asian	K/L score	TaqMan	PB	725	1737
2014	Suliang Lou	Knee OA	China	Asian	K/L score	TaqMan	PB	152	179
2015	LinWang	Knee OA	China	Asian	K/L score	iMLDR	PB	164	200
2016	Thitiya Poonpet	Knee OA	Thai	Asian	K/L score	HRM analysis	PB	200	200

*K/L score* Kellgren–Lawrence score, *PB* population based, *TKR* total knee replacement, *TFOA* tibiofemoral knee OA, *PCR-RFLP* polymerase chain reaction-restriction fragment length polymorphism, *PFOA* patellofemoral knee OA, *iMLDR* improved multiplex ligase detection reaction, *HRM* high resolution melting

**Table 2:** Distributions of ADAM2 rs3740199 genotypes and alleles among cases and controls

Year	First author	Case			Control			Case		Control		HWE in control
		CC	GC	GG	CC	GC	GG	C	G	C	G	
2009	J. Rodriguez-Lopez^a^	NA	NA	NA	NA	NA	NA	290	234	327	261	NA
2009	J. Rodriguez-Lopez^a^	NA	NA	NA	NA	NA	NA	370	350	744	652	NA
2009	J. Rodriguez-Lopez^a^	NA	NA	NA	NA	NA	NA	180	138	239	147	NA
2009	I. Kerna^a^	28	32	6	65	46	12	88	44	176	70	0.366
2009	I. Kerna^a^	53	34	10	41	43	8	140	54	125	59	0.485
2012	Min-Ho Shin	147	364	214	350	863	524	658	792	1563	1911	0.876
2014	Suliang Lou	32	78	42	42	93	44	142	162	177	181	0.6
2015	LinWang	36	84	44	47	102	51	156	172	196	204	0.773
2016	Thitiya Poonpet	56	102	42	46	100	54	214	186	192	208	0.982

*HWE* Hardy–Weinberg equilibrium, *NA* data not available

^a^An independent study in one article

### Quantitative synthesis

The details of meta-analysis for *ADAM12* rs3740199 polymorphism with knee OA risk are shown in Table 3.

**Table 3:** Meta-analysis for the ADAM2 rs3740199 polymorphism and knee OA risk

Population	Comparison (*N* ^a^)	Test of association		Test of heterogeneity	
		OR (95% CI)	*P* ^b^	*P* ^c^	*I* ^2^ (%)
Overall	C vs. G (12)	0.983 (0.910–1.061)	0.657	0.490	0.0
	CC vs. GG (6)	1.033 (0.851–1.255)	0.740	0.681	0.0
	CG vs. GG (6)	1.030 (0.877–1.209)	0.721	0.770	0.0
	CC/CG vs. GG (6)	1.031 (0.886–1.201)	0.690	0.768	0.0
	CC vs. CG/GG (6)	1.017 (0.868–1.190)	0.837	0.364	8.1
Ethnicity					
Asian	C vs. G (4)	1.020 (0.924–1.127)	0.689	0.398	0.0
	CC vs. GG (4)	1.040 (0.850–1.272)	0.703	0.390	0.4
	CG vs. GG (4)	1.035 (0.878–1.221)	0.681	0.709	0.0
	CC/CG vs. GG (4)	1.036 (0.887–1.211)	0.656	0.502	0.0
	CC vs. CG/GG (4)	1.017 (0.858–1.207)	0.843	0.642	0.0
European	C vs. G (8)	0.930 (0.825–1.049)	0.239	0.538	0.0
	CC vs. GG (2)	0.950 (0.453–1.992)	0.891	0.809	0.0
	CG vs. GG (2)	0.927 (0.446–1.930)	0.840	0.301	6.6
	CC/CG vs. GG (2)	0.940 (0.465–1.901)	0.864	0.713	0.0
	CC vs. CG/GG (2)	0.998 (0.445–2.237)	0.996	0.052	73.4

*OR* odds ratio, *CI* confidence interval

^a^Number of comparison

^b^ *P* values for within group differences were determined by *Z* test

^c^ *P* value of *Q* test for heterogeneity test

### Overall population

Nine separate studies with a total sample size of 2185 cases and 3716 controls had available data for analyzing the association of *ADAM12* rs3740199 polymorphism and knee OA risk. No significant association was found in all genetic models (C vs. G, OR = 0.983, 95% CI = 0.910–1.061; CC vs. GG, OR = 1.033, 95% CI = 0.851–1.255; CG vs. GG, OR = 1.030, 95% CI = 0.877–1.209; CC/CG vs. GG, OR = 1.031, 95% CI = 0.886–1.201; CC vs. qECG/GG, OR = 1.017, 95% CI = 0.868–1.190) (Table 3).

### Subgroup analysis by ethnicity

No significant association was found in all genetic models among Asian population (C vs. G, OR = 1.020, 95% CI = 0.924–1.127; CC vs. GG, OR = 1.040, 95% CI = 0.850–1.272; CG vs. GG, OR = 1.035, 95% CI = 0.878–1.221; CC/CG vs. GG, OR = 1.036, 95% CI = 0.887–1.211; CC vs. CG/GG, OR = 1.017, 95% CI = 0.858–1.207). No significant association was found in European population either.

### Heterogeneity and publication bias

The between-study heterogeneity of the *ADAM12* rs3740199 polymorphism was not found in all subjects, and thus, the meta-analysis of the *ADAM12* rs3740199 polymorphism was performed using a fixed-effects model for all subjects except for the recessive model in European population, which was analyzed using a random-effects model (*I* ^2^ = 73.4%) (Table 3).

**Fig. 2:**
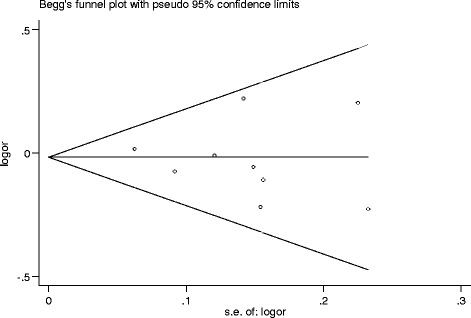
Begg’s funnel plot for publication bias test (C versus G). Each point represented a separate study for the indicated association. *logor* natural logarithm of OR. *Horizontal line* mean effect size

In this study, publication bias was evaluated by funnel plot and the linear regression asymmetry test. As shown in Fig. 2, the shape of the funnel did not reveal obvious asymmetry. Then, Egger’s test was then performed to estimate the funnel plot symmetry. The results still did not show any evidence of publication bias (*t* = −0.40, *P* = 0.699 for C vs. G).

## Discussion

OA is well recognized as a multifactorial disease. In addition to age, sex, trauma, and body weight, genetic factors are also strong determinants of this disease [30]. More than 50% of the OA cases can be attributed to genetic factors, demonstrated by twins and family studies [31]. It is suggested that OA of the hand, spine, hip, and knee are all heritable [32]. Recently, genetic studies have found many genes are contributing to OA, although with relatively modest effect [33]. These observations have encouraged us to search for more responsible genes. Many genes have been studied and the *ADAM12* gene is one of the possible candidate genes for OA [12, 23–27]. Some investigations suggested a regulatory role of *ADAM12* in chondrocyte proliferation, maturation, bone formation, and osteoclast differentiation [15–18]. Association studies have been arranged to investigate the role of a nonsynonymous polymorphism (rs3740199) in the second exon of *ADAM12* in knee OA risk that has been reported to date [12, 21, 25, 26, 34]. However, these findings have been inconsistent and contradictory.

Meta-analysis is a suitable method to combine the results of individual studies, overcome the disadvantages of a single study, and increase the statistical power. The present study was to investigate and update the results associating the *ADAM12* rs3740199 polymorphism with knee OA risk in different ethnic populations. To our knowledge, the present study is the first meta-analysis which estimated the association between *ADAM12* rs3740199 and knee OA susceptibility. No significant association of *ADAM12* rs3740199 polymorphisms with knee OA risk was demonstrated in our study. We also failed to find the association between knee OA and the *ADAM12* polymorphism in Asian and European population. No heterogeneity was found in overall population and Asian population, while high heterogeneity was seen in the recessive model in European population.

Association studies with complex outcomes for detecting genetic variants must be considered with caution because the results may be influenced by many factors. Our present study showed a lack of association between the *ADAM12* rs3740199 polymorphism and knee OA risk, which is not consistent with the association or functional studies of the *ADAM12* polymorphism. However, epidemiologic results are always different from the functional studies because OA is a multifactorial disease influenced by different genetic backgrounds, multiple genes, and environmental factors. Our negative results of the *ADAM12* polymorphism may also be due to type II error. In recent years, some genome-wide association studies (GWAS) have already reported *GDF5*, *BTNL2*, *DUS4L*, *COG5*, *SENP6*, and *FILIP1* as OA candidate genes [35, 36]. However, the *ADAM12* polymorphism has not been confirmed in these GWAS [35, 36].

Our results are consistent with some studies that also failed to detect association between rs3740199 and OA susceptibility in either male or female patients [12, 23, 26, 27]. On the other hand, Poonpet et al. reported the rs3740199 polymorphism was associated with knee OA risk, while the effect was only found in Thai male patients [24]. The C allele of rs3740199 was found to be associated with OA in female patients in the UK [21]. Kerna et al. found significant association between *ADAM12* rs3740199 and PFOA in male patients [25]. The reason for the contradictory results remain unclear, but the differences in study populations including age, gender, sample size, and disease severity may play an important role. In the meanwhile, the environmental factors in each sample such as lifestyles, diets, and selected physical activity may also affect the association between rs3740199 polymorphism and OA risk. Lastly, each population has their own gene pool, so it is not surprising that there are differences in the distributions of *ADAM12* rs3740199 genotypes and alleles from subjects with different ethnicities.

Some limitations of this meta-analysis should also be noted. First, the potential confounding factors (such as age, gender) were not adjusted in the present study. Second, the gene-environment interactions and the effect of gene-gene should also be considered because they might influence the biological effects of the polymorphisms of the *ADAM12* gene. Third, because it was difficult to get all full papers published in different languages, we only included six studies published in English language. Fourth, the included subjects were not adequate to confirm a robust conclusion and the association should be resolved by larger studies.

## Conclusions

In conclusion, this meta-analysis did not reveal any association between the *ADAM12* rs3740199 polymorphism and knee OA risk. Additional larger studies are needed to confirm our findings in the future.

## Abbreviations

ADAM: A disintegrin and metalloprotease
CIs: Confidence intervals
GWAS: Genome-wide association studies
HRM: High resolution melting
HWE: Hardy–Weinberg equilibrium
iMLDR: Improved multiplex ligase detection reaction
K/L score: Kellgren–Lawrence score
OA: Osteoarthritis
ORs: Odds ratios
PB: Population based
PCR-RFLP: Polymerase chain reaction-restriction fragment length polymorphism
PFOA: Patellofemoral knee OA
TFOA: Tibiofemoral knee OA
TKR: Total knee replacement
